# Alleviating the hypoxic tumor microenvironment with MnO_2_-coated CeO_2_ nanoplatform for magnetic resonance imaging guided radiotherapy

**DOI:** 10.1186/s12951-023-01850-1

**Published:** 2023-03-15

**Authors:** Fen Pi, Xuanru Deng, Qian Xue, Lan Zheng, Hongxing Liu, Fang Yang, Tianfeng Chen

**Affiliations:** 1 Department of Chemistry, Guangdong Provincial Key Laboratory of Functional Supramolecular Coordination Materials and Applications, Jinan University, Guangzhou, 510632 China; 2 Department of Urology, Guangzhou Institute of Urology, Guangdong Key Laboratory of Urology, The First Affiliated Hospital of Guangzhou Medical University, Guangzhou, China

**Keywords:** Radiosensitizer, Hypoxia, Core–shell nanocomposite, Antitumor, MR imaging

## Abstract

**Background:**

Radiotherapy is a commonly used tool in clinical practice to treat solid tumors. However, due to the unique microenvironment inside the tumor, such as high levels of GSH, overexpressed H_2_O_2_ and hypoxia, these factors can seriously affect the effectiveness of radiotherapy.

**Results:**

Therefore, to further improve the efficiency of radiotherapy, a core–shell nanocomposite CeO_2_–MnO_2_ is designed as a novel radiosensitizer that can modulate the tumor microenvironment (TME) and thus improve the efficacy of radiation therapy. CeO_2_–MnO_2_ can act as a radiosensitizer to enhance X-ray absorption at the tumor site while triggering the response behavior associated with the tumor microenvironment. According to in vivo and in vitro experiments, the nanoparticles aggravate the killing effect on tumor cells by generating large amounts of ROS and disrupting the redox balance. In this process, the outer layer of MnO_2_ reacts with GSH and H_2_O_2_ in the tumor microenvironment to generate ROS and release oxygen, thus alleviating the hypoxic condition in the tumor area. Meanwhile, the manganese ions produced by degradation can enhance T1-weighted magnetic resonance imaging (MRI). In addition, CeO_2_–MnO_2_, due to its high atomic number oxide CeO_2_, releases a large number of electrons under the effect of radiotherapy, which further reacts with intracellular molecules to produce reactive oxygen species and enhances the killing effect on tumor cells, thus having the effect of radiotherapy sensitization. In conclusion, the nanomaterial CeO_2_–MnO_2_, as a novel radiosensitizer, greatly improves the efficiency of cancer radiation therapy by improving the lack of oxygen in tumor and responding to the tumor microenvironment, providing an effective strategy for the construction of nanosystem with radiosensitizing function.

**Conclusion:**

In conclusion, the nanomaterial CeO_2_–MnO_2_, as a novel radiosensitizer, greatly improves the efficiency of cancer radiation therapy by improving the lack of oxygen in tumor and responding to the tumor microenvironment, providing an effective strategy for the construction of nanosystems with radiosensitizing function.

**Supplementary Information:**

The online version contains supplementary material available at 10.1186/s12951-023-01850-1.

## Introduction

Cancer is one of the most life-threatening diseases to human health [1]. Researchers have developed various anti-cancer strategies such as chemotherapy, radiotherapy and immunotherapy [2–4]. Among them, radiotherapy is a very effective and commonly used method to eliminate tumors [5]. However, the rapid growth of tumors leads to tumor microenvironment characterized by hypoxia, microacidity, high levels of glutathione and hydrogen peroxide [6–8], which also makes radiation therapy less effective and creates radiotherapy resistance.

Moreover, in clinical practice, radiotherapy inevitably causes irreversible damage to normal tissues and cells [9–11]. Therefore, the development of radiotherapy sensitizers can greatly overcome the shortcomings of conventional radiotherapy and reduce the toxic side effects caused by conventional radiotherapy [12–14]. Currently, it has been found that many materials have the ability to enhance the sensitivity of tumor cells to X-rays, such as the small molecule paclitaxel [15–17] and metal complexes [18–21]. However, they are less selective and more toxic to normal cells and tissues. Therefore, there is an urgent need for researchers to develop new efficient and low-toxic radiotherapy sensitizers.

Researchers have studied and developed numerous novel radiotherapy sensitizers, with nanomaterials being particularly prominent [22–24]. Nanomaterials usually respond to the tumor microenvironment and can boost the sensitivity of tumor tissue to radiation [25]. They can increase the radiotherapy effect at the lesion site and achieve radiotherapy sensitization. Metal nanoparticles with high atomic number are introduced into tumor tissues and then treated with high-energy radiation to release electrons [26, 27]. The released electrons react with organic molecules or water in cancer cells to produce large amounts of ROS, thus enhancing the effect of radiotherapy [28–30]. However, without metal nanoparticles of high atomic number, the effect of radiotherapy sensitization is not satisfactory. As a metal oxide with a high atomic number, CeO_2_ can enhance the deposition of intracellular radiation and produce a large amount of free radicals to kill tumor cells in the presence of X-rays [31, 32]. At the same time, it has low toxicity to normal tissues and cells, which can overcome the toxic and side effects caused by conventional radiotherapy [33, 34].

Hypoxia is a prominent feature of the tumor microenvironment and has long been considered as a key factor contributing to the tolerance of radiotherapy in solid tumors [35–37]. In recent years, researchers have also developed different strategies to alleviate hypoxia within the tumor, such as oxygen delivery to the tumor region [38, 39] and in situ oxygen generation [40–42] in the tumor region. However, there is a problem with the strategy of delivering oxygen to the hypoxic region in a tumor due to the uneven distribution of blood vessels within the tumor. To solve the above problem, the high H_2_O_2_ concentration in the tumor region has been used to catalyze the in situ generation of oxygen. MnO_2_ nanomaterials have proven to be a hot spot for researchers who are seeking to catalyze the production of O_2_ from H_2_O_2_ to overcome the problem of tumor hypoxia [43, 44]. Moreover, Mn^2+^ generated by the reaction between MnO_2_ and GSH can be used in MRI [45, 46]. Therefore, the radiation therapy effect can be enhanced by making full use of the radiotherapy sensitizing property of CeO_2_ and the property of MnO_2_ to improve the hypoxic condition in the tumor area and enhance the radiotherapy effect. Compared to traditional radiotherapy sensitizers, the nanoparticles we proposed have the advantages of high efficiency and low toxicity, high selectivity and guided treatment by MRI.

**Scheme 1:**
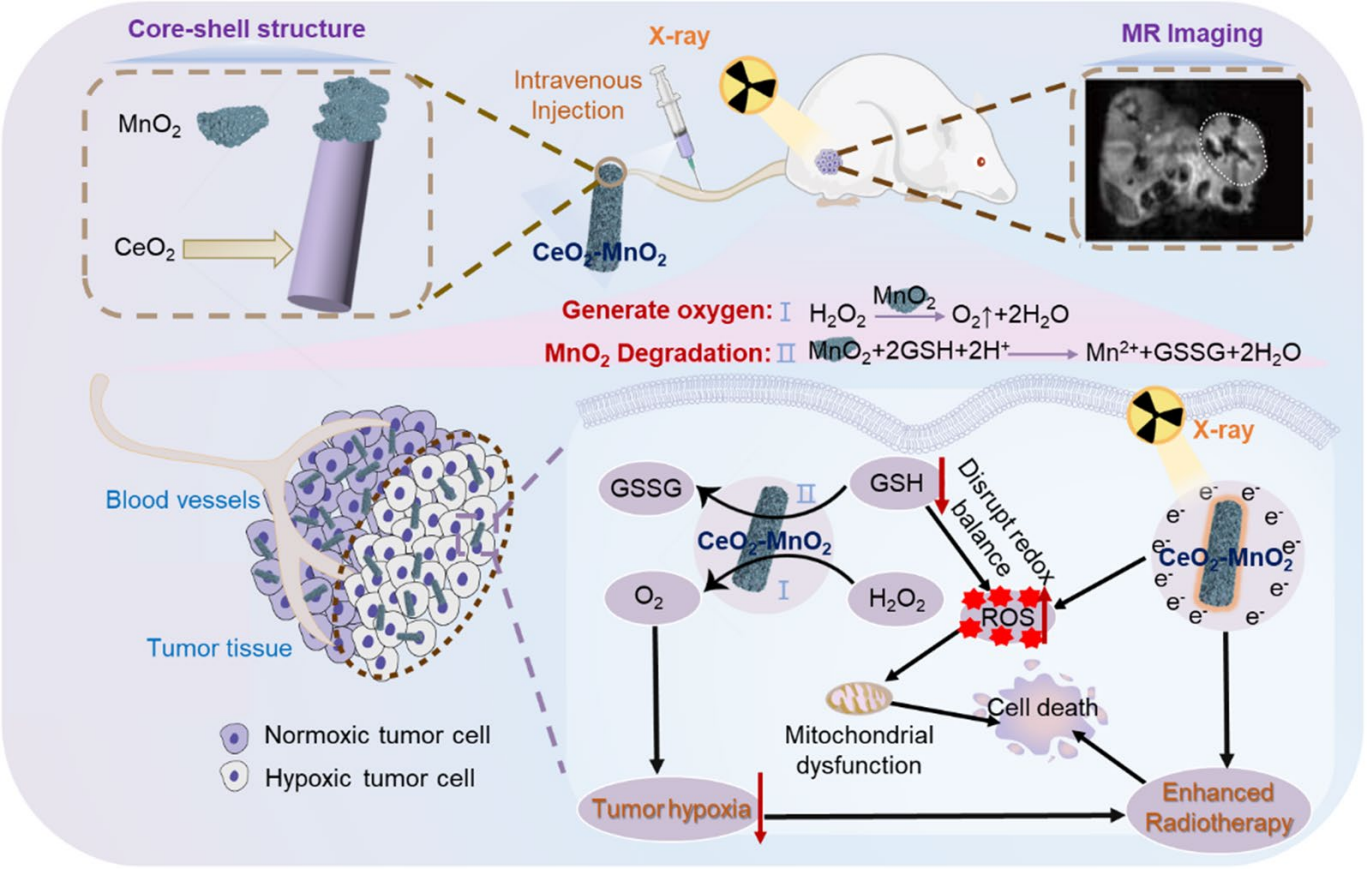
Schematic structure of CeO_2_-MnO_2_ and its synergistic mechanism for the treatment of hypoxic tumors

The goal of this study was to synthesize core–shell CeO_2_–MnO_2_ nanoparticles with significant radiosensitizing effect by hydrothermal method [47], which was found to be effective in killing solid tumors and improving tumor hypoxia. First, the successful synthesis of core–shell CeO_2_–MnO_2_ nanoparticles was demonstrated by a series of characterization methods. Subsequently, it was proven in vitro that CeO_2_–MnO_2_ has superior performance in catalyzing the generation of O_2_ from hydrogen peroxide. Finally, using MIHA cells as a normal cell model, the synergistic group of CeO_2_–MnO_2_ and X-ray was confirmed to have a significant protective effect on normal cells by MTT assays. HeLa cells were also used as a tumor cell model, and in vivo and in vitro experiments suggested that under X-ray irradiation, CeO_2_–MnO_2_ exerted a positive anti-tumor effect by generating massive ROS in the cells, leading to a flip in mitochondrial membrane potential and accelerating apoptosis of tumor cells (Scheme 1). In conclusion, CeO_2_–MnO_2_ nanoparticle is a novel, low-toxicity radiosensitizing nanosystem that improves the efficiency of radiation therapy in vivo and in vitro by improving hypoxia, enhancing ROS production and promoting apoptosis of cancer cells.

## Results and discussion

### Rational design and synthesis of CeO_2_–MnO_2_ nanosystem

**Fig. 1:**
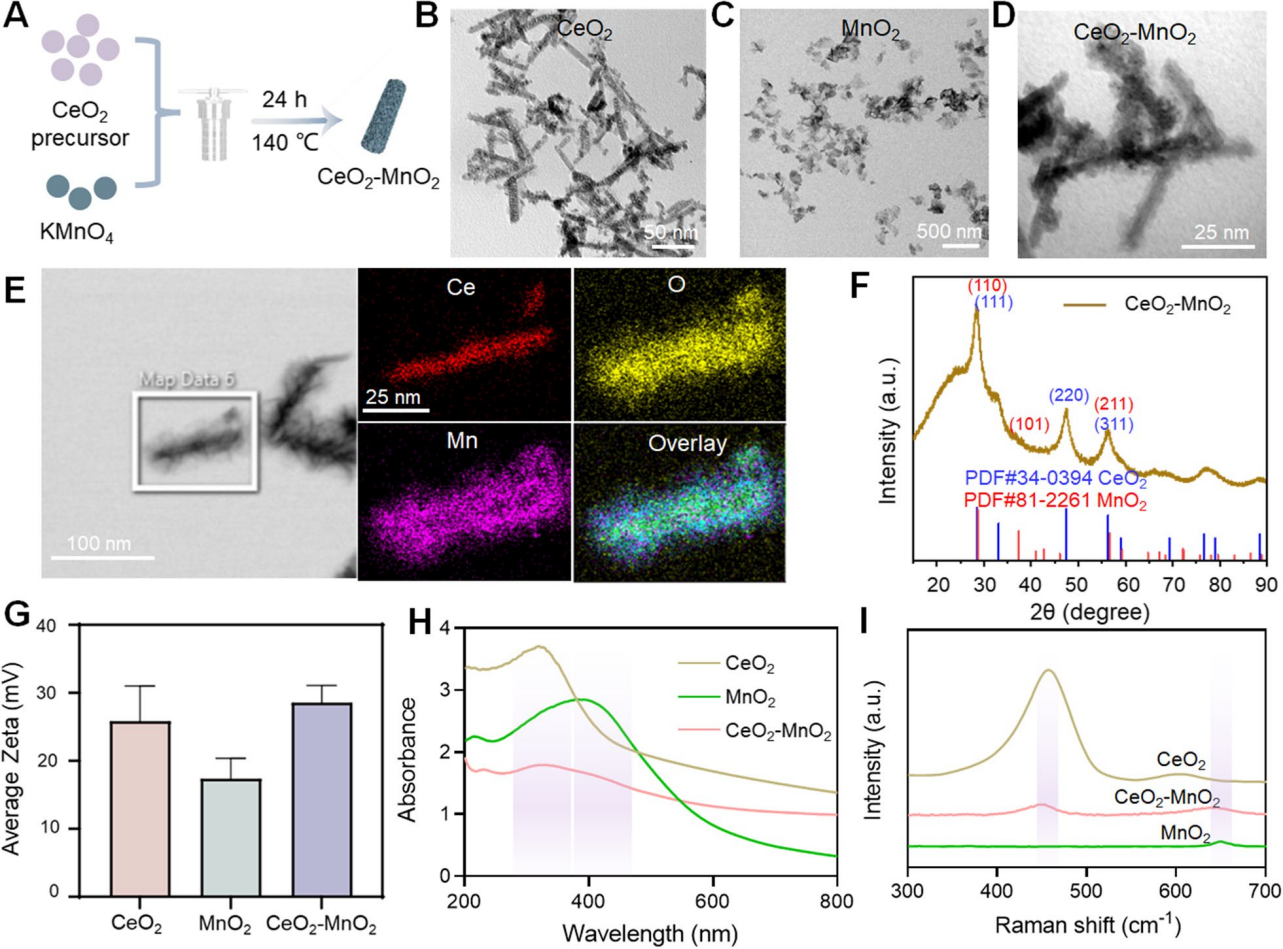
Synthesis and characterization of CeO_2_–MnO_2_. **A** Diagrams for synthetic process of CeO_2_–MnO_2_. **B** TEM images of CeO_2_. Scale bar = 50 nm. **C** TEM images of MnO_2_. Scale bar = 500 nm. **D** TEM images of CeO_2_–MnO_2_. Scale bar = 25 nm. **E** EDS element mapping images of CeO_2_–MnO_2_. Scale bar = 25 nm. **F** XRD analysis of CeO_2_, MnO_2_ and CeO_2_–MnO_2_. **G** The average zeta of CeO_2_, MnO_2_ and CeO_2_–MnO_2_. **H** The UV spectra of CeO_2_, MnO_2_ and CeO_2_–MnO_2_ with different concentrations. **I** The Raman diagram of CeO_2_, MnO_2_ and CeO_2_–MnO_2_

In this study, we synthesized CeO_2_–MnO_2_ nanoparticles through using hydrothermal method (Fig. 1A). The size and shape of the materials were investigated by TEM. Figure 1B showed that CeO_2_ was a rod-like nanoparticle with a particle size of about 100 nm. MnO_2_ (Fig. 1C) was a nanoparticle that exhibits a distinct sheet-like shape with a particle size of about 150 nm. It is obvious that CeO_2_–MnO_2_ displays a core–shell structure with a size of about 100 nm. Rod-shaped CeO_2_ nanoparticles were covered with MnO_2_ nanosheets (Fig. 1D). Based on the EDS elemental analysis (Fig. 1E), the above conclusion can also be verified. Mn and O elements were observed on the surface of CeO_2_, further verifying the encapsulation of MnO_2_ on CeO_2_. The hydration diameters (Additional file 1: Fig. S1) and potential diagrams (Fig. 1G) showed that the average hydration diameter of CeO_2_ nanoparticles is about 450 nm, and MnO_2_ is about 120 nm, and the combined CeO_2_–MnO_2_ is about 580 nm. CeO_2_ and MnO_2_ alone have obvious positive electrical properties, and CeO_2_–MnO_2_ exhibits stronger positive electrical properties. Besides, to further evaluate the encapsulation of MnO_2_ on the CeO_2_ surface, Raman, UV and XRD analyses were performed. According to the Raman diagram (Fig. 1I), it was observed that CeO_2_–MnO_2_ nanoparticles have common peaks with CeO_2_ and MnO_2_ at about 460 cm^−1^ and 670 cm^−1^, respectively. The presence of CeO_2_ and MnO_2_ in CeO_2_–MnO_2_ nanoparticles were verified by UV–Vis spectroscopy (Fig. 1H). Also, the results demonstrate the CeO_2_–MnO_2_ have the same peaks with CeO_2_ and MnO_2_ respectively, corresponding to 123 nm and 399 nm. X-ray diffraction (XRD) patterns showed that the characteristic peaks of CeO_2_, MnO_2_ all corresponded to CeO_2_–MnO_2_, in accordance with the JCPDS No. 81-2261 of the MnO_2_ crystal and JCPDS No. 34-0394 of the CeO_2_ crystal (Fig. 1F). In summary, all results confirm the successful synthesis of CeO_2_–MnO_2._

### The ability of CeO_2_–MnO_2_ to catalyze hydrogen peroxide, depletion of GSH, rise in ROS concentration

**Fig. 2:**
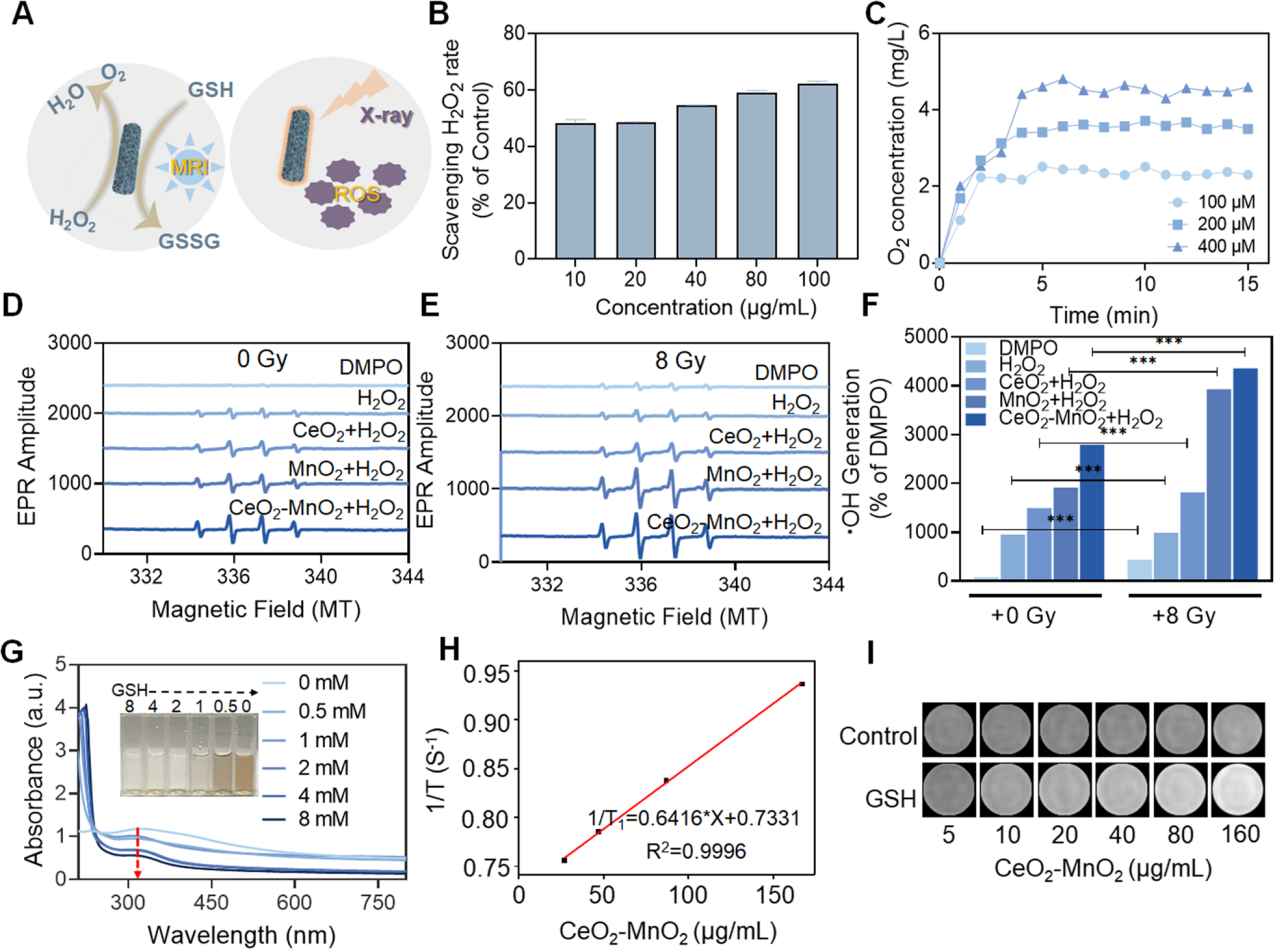
The ability of CeO_2_–MnO_2_ to catalyze hydrogen peroxide, depletion of GSH, rise in ROS induced by radiotherapy, and MRI properties. **A** Schematic diagram of CeO_2_–MnO_2_ catalyze hydrogen peroxide, depletion of GSH and promotion of radiotherapy-induced ROS rise and in vitro imaging. **B** Rates of hydrogen peroxide scavenging by CeO_2_–MnO_2_ under different concentrations. **C** The amount of O_2_ catalyzed by co-incubation of CeO_2_–MnO_2_ with 0.1 μg/mL hydrogen peroxide for 15 min. **D** ESR analysis of •OH production of CeO_2_, MnO_2_ and CeO_2_–MnO_2_. (E) ESR analysis of •OH production of CeO_2_, MnO_2_ and CeO_2_–MnO_2_ under X-ray (8 Gy). **F** Quantification of •OH production rate in the presence (8 Gy) and absence of radiotherapy. **G** UV absorption of CeO_2_–MnO_2_ after interaction with different concentrations of GSH and pictures of CeO_2_–MnO_2_ after interaction with different concentrations of GSH. **H** T1 relaxation rate associated with CeO_2_–MnO_2_ concentration in the presence of GSH. **I** T1-weighted photographs of different concentrations of CeO_2_–MnO_2_ in the presence or absence of GSH

The catalysis of hydrogen peroxide by CeO_2_–MnO_2_, the depleted GSH, as well as the rise in ROS induced by radiotherapy are shown in Fig. 2A. Hypoxia leads to the insensitivity of tumor cells to radiotherapy. To verify that CeO_2_–MnO_2_ nanoparticles have favorable catalytic properties to produce oxygen from hydrogen peroxide, the rate of hydrogen peroxide scavenging by CeO_2_–MnO_2_ was examined in vitro using a hydrogen peroxide kit. The results showed that CeO_2_–MnO_2_ had the fastest hydrogen peroxide clearance at a concentration of 100 μg/mL compared to CeO_2_ and MnO_2_ alone, with a clearance rate of approximately 60% compared to the control group (Additional file 1: Fig. S2). The rate of H_2_O_2_ scavenging by CeO_2_–MnO_2_ at different concentrations was also examined, and Fig. 2B showed that the scavenging rate had a significant concentration dependence. To further investigate the performance of the material to catalyze the generation of oxygen from hydrogen peroxide, we monitored the ability to generate oxygen within 15 min by adding different concentrations of H_2_O_2_ to the CeO_2_–MnO_2_ solution using a dissolved oxygen analyzer. Figure 2C showed that the oxygen content reached a maximum after 5 min, and the amount of O_2_ produced was dependent on the concentration of H_2_O_2_. These results indicate that CeO_2_–MnO_2_ has a reasonable ability to catalyze the production of O_2_ from H_2_O_2_. Radiotherapy can lead to the deposition of intracellular energy and the generation of large amounts of reactive oxygen species. These reactive oxygen species can disrupt the redox balance in cells and thus can lead to cellular damage. Therefore, we next explored the overproduction of ROS triggered by CeO_2_–MnO_2_ combined with X-ray irradiation. Electron spin resonance (ESR) spectroscopy results confirm that CeO_2_–MnO_2_ enhances •OH production, while X-ray (8 Gy) irradiation further increases •OH production (Fig. 2D, F). We also used DCFH-DA and DHE probes to detect ROS and •O_2_^−^ generated before and after CeO_2_–MnO_2_ combined with X-ray (Additional file 1: Fig. S3–S4). Although CeO_2_–MnO_2_ was also able to produce ROS in the absence of X-rays, the ROS level increased significantly after combining with X-ray, and it was higher than that of the CeO_2_ combined X-ray group and MnO_2_ combined X-ray group. The above results indicate that CeO_2_–MnO_2_ combined with X-ray can produce a large amount of ROS. The content of GSH inside the tumor is higher than that of normal cells. Due to the fact that GSH scavenges free radicals to protect cells, the overexpression of GSH can reduce the effects of radiotherapy. Therefore, the responsiveness of CeO_2_–MnO_2_ to GSH was investigated. It is shown in Fig. 2G that the characteristic absorption peaks of CeO_2_–MnO_2_ in the UV spectrum decreased with the increase of GSH concentration, indicating the reaction of both. Additionally, the color of CeO_2_–MnO_2_ solution changed from yellow to colorless as GSH concentration increased, indicating that CeO_2_–MnO_2_ consumed GSH. T1-weighted MRI signal may be enhanced by CeO_2_–MnO_2_ since it is capable of consuming GSH in TME and generating Mn^2+^. Therefore, we evaluated the imaging capability of CeO_2_–MnO_2_ in vitro. As shown in Fig. 2H, I, the T1-weighted signal intensity of CeO_2_–MnO_2_ was significantly enhanced in the presence of GSH. As a result, CeO_2_–MnO_2_ is decomposed by GSH to generate Mn^2+^, which enhances the T1-weighted signal. The above results reveal that CeO_2_–MnO_2_ nanoparticles have superior functions of enhancing ROS, catalyzing oxygen generation from hydrogen peroxide.

### X-rays stimulate ROS production to enhance the anti-cancer effect of CeO_2_–MnO_2_

**Fig. 3:**
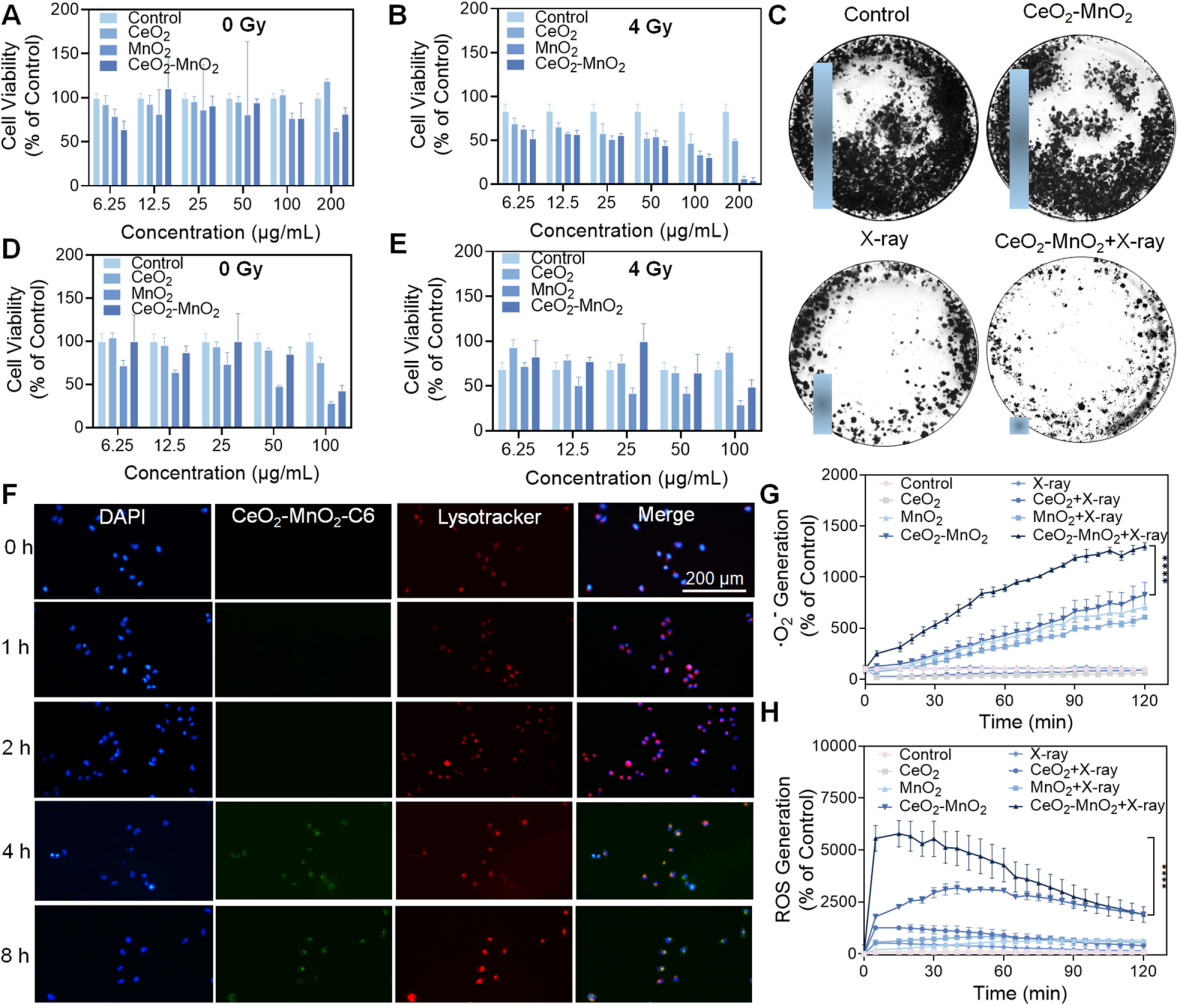
ROS are generated by X-rays in a manner that synergistically enhances the anti-cancer efficacy of CeO_2_–MnO_2_. **A** The cell viability of HeLa cells treated by CeO_2_, MnO_2_ and CeO_2_–MnO_2_. **B** The cell viability of HeLa cells stimulated by CeO_2_, MnO_2_ and CeO_2_–MnO_2_ under X-ray (4 Gy). **C** Colony formation experiment of HeLa cells subjected to different treatments. **D** The cell viability of MIHA cells treated by CeO_2_, MnO_2_ and CeO_2_–MnO_2_. **E** The cell viability of MIHA cells induced by CeO_2_, MnO_2_ and CeO_2_–MnO_2_ under X-ray (4 Gy). **F** Co-localization of CeO_2_–MnO_2_ with HeLa cells. **G** •O_2_^−^ level of HeLa cells after treatment with different groups and X-rays. **H** ROS level of HeLa cells after treatment with different drug groups and X-rays

In order to investigate the sensitizing effect of CeO_2_–MnO_2_ nanoparticles for radiotherapy, HeLa cells were used as model cancer cells in vitro, and CeO_2_–MnO_2_ CeO_2_–MnO_2_ was co-incubated with HeLa cells to detect their cell survival rate. Figure 3A showed that the CeO_2_–MnO_2_ treatment group had toxic effects on HeLa cells. We further investigated the antitumor effect of CeO_2_–MnO_2_ combined with X-rays in vitro. Figure 3B illustrated that the combination of CeO_2_–MnO_2_ with X-rays showed enhanced cytotoxicity when compared to the X-ray group alone, as well as stronger cytotoxicity than either the CeO_2_ or MnO_2_ groups alone. By analyzing the interaction between the concentration of CeO_2_–MnO_2_ and the X-ray dose, the results were obtained by isobologram analysis. Additional file 1: Fig. S5 showed that CeO_2_–MnO_2_ has a significant radiotherapy sensitizing effect under 4 Gy. Subcellular localization experiments showed that coumarin 6-labeled CeO_2_–MnO_2_ (green fluorescence) could effectively enter HeLa cells after 4 h and that lysosomes were the main organelle targets of CeO_2_–MnO_2_ (Fig. 3F). Since radiotherapy leads to toxic effects on normal cells and tissues, the development of safe and non-toxic radiotherapy sensitizers is an urgent issue. To evaluate the radiation protection effect of CeO_2_–MnO_2_ on normal cells, we determined the cellular activity of MIHA (human normal hepatocytes) after CeO_2_–MnO_2_ combined with 4 Gy using the MTT assay. As shown in Fig. 3D, E, the cell survival rate decreased as the concentration of each drug increased in the absence of radiation irradiation. When MIHA cells were irradiated with 4 Gy, the cell survival rate in the CeO_2_–MnO_2_ group was higher than that in the X-ray alone group. However, when the concentration of CeO_2_–MnO_2_ reached 100 μg/mL, the cell survival rate decreased after the combined action with X-rays, which might be due to the toxicity of the drug dose. According to the above results, CeO_2_–MnO_2_ concentrations below 100 μg/mL may have some radiation protection effect on normal cells. CeO_2_–MnO_2_ was found to have a significant synergistic effect with X-ray in inhibiting HeLa cells using colony formation experiments (Fig. 3C). These results suggest that CeO_2_–MnO_2_ can be used as a radiation therapy sensitizer combined with X-rays to inhibit the growth of HeLa cells. High levels of ROS disrupt the intracellular redox balance and will enhance the biomolecular damage induced by ionizing radiation, which is the main mechanism by which CeO_2_–MnO_2_ enhances the effect of radiotherapy. As shown in Fig. 3G, H, CeO_2_–MnO_2_ increased the accumulation of ROS and •O_2_^−^ in HeLa cells under X-ray treatment. Therefore, it can be concluded that CeO_2_–MnO_2_ may significantly enhance radiotherapy damage of HeLa cells by enhancing the production of ROS, thus exhibiting superior antitumor effects in vitro.

### CeO_2_–MnO_2_ combined with X-ray regulates mitochondrial damage, cell cycle and apoptosis

**Fig. 4:**
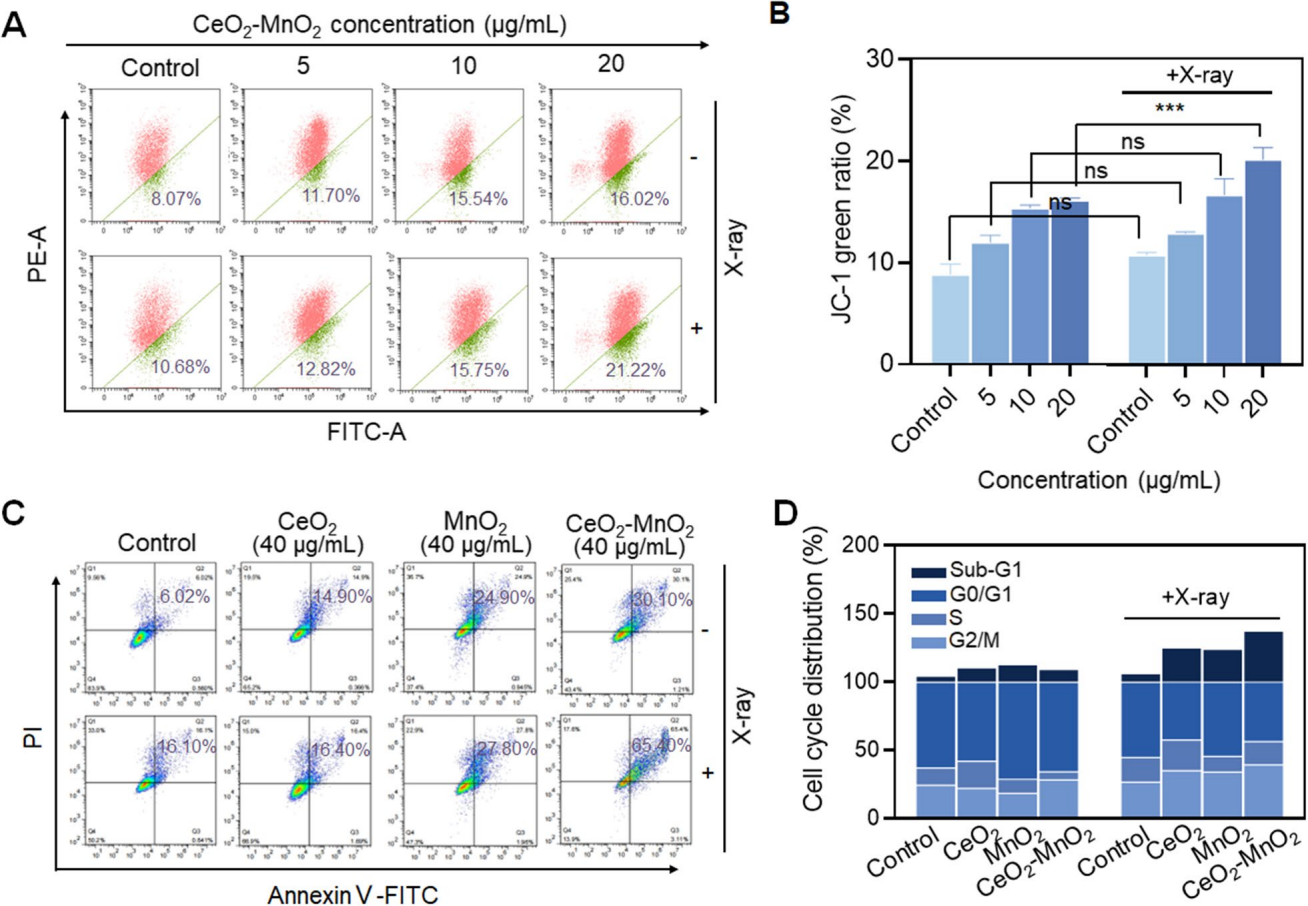
CeO_2_–MnO_2_ combined with X-ray regulates mitochondrial damage, cell cycle and apoptosis. **A** Mitochondrial membrane potential in different concentration of CeO_2_–MnO_2_ and X-rays (4 Gy). **B** Quantitative analysis of the proportion of the JC-1 green ratio with or without radiation (4 Gy) under the same concentration of CeO_2_–MnO_2_ in HeLa cells. **C** Cell apoptosis analysis of HeLa cells exposed to 40 µg/mL CeO_2_, MnO_2_, and CeO_2_–MnO_2_ under different X-rays (4 Gy). **D** Cell-cycle quantitative analysis after different treatments was detected using PI staining

Elevated ROS levels can lead to an imbalance in cellular redox homeostasis, resulting in mitochondrial dysfunction, which further induces cell damage and apoptosis. We first examined the changes in mitochondrial membrane potential (MMP, Δψm) in HeLa cells triggered by the combination of different concentrations of CeO_2_–MnO_2_ and X-rays (4 Gy) using the JC-1 probe. As shown in Fig. 4A, CeO_2_–MnO_2_ caused a slight decrease in mitochondrial membrane potential, and the change in mitochondrial membrane potential was more pronounced and concentration-dependent after combined with X-ray irradiation. This can be seen in the green fluorescence ratio (Fig. 4B). It was discovered that CeO_2_–MnO_2_ has a radiosensitizing effect. However, the percentage of apoptosis was significantly elevated after CeO_2_–MnO_2_ combined with X-ray treatment, further indicating the radiosensitizing effect of CeO_2_–MnO_2_. The percentage of apoptotic cells after treatment with different drug groups was detected using an apoptosis kit. Figure 4C showed that treatment of HeLa cells with CeO_2_–MnO_2_ and X-rays induced mainly late-stage apoptosis. The late-stage apoptosis rate increased gradually from 6.02 (control) to 30.10% after treatment with CeO_2_–MnO_2_, and further increased to 65.40% after combined with X-ray irradiation. And the effect of CeO_2_–MnO_2_ on triggering late-stage apoptosis was more significant compared to CeO_2_ and MnO_2_ alone. Furthermore, we analyzed the effect of CeO_2_–MnO_2_ combined with X-ray on the HeLa cell cycle using flow cytometry. Figure 4D shows that the group of CeO_2_–MnO_2_ combined with X-ray mainly caused elevated Sub-G1 phase in HeLa cells. These results suggest that CeO_2_–MnO_2_ can effectively enhance X-ray-induced mitochondrial damage and ultimately promote apoptosis.

### Therapeutic effect of CeO_2_–MnO_2_ and MR imaging in vivo

**Fig. 5:**
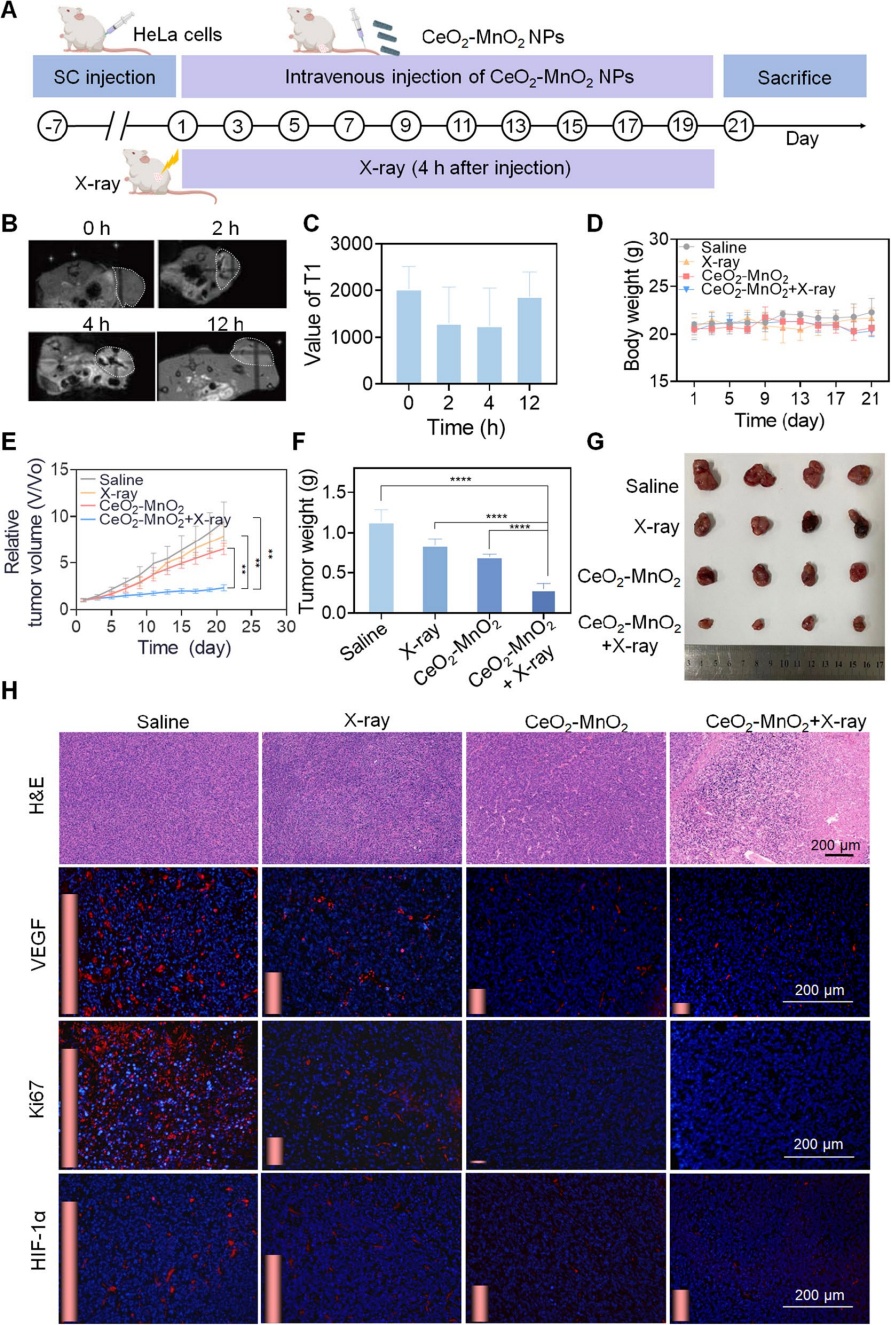
In vivo antitumor effect of CeO_2_–MnO_2_ combined with X-ray. **A** Schematic diagram of the animal experiment. **B** In vivo T1-weighted MRI images of tumor-bearing mice after intravenous injection of CeO_2_–MnO_2_ at different periods. **C** T1 values of tumor-bearing mice after intravenous injection of CeO_2_–MnO_2_ at different periods. **D** The body weight during 21 days treatment. **E** Tumor relative volume curves during 21 days. **F** Relative tumor weight after 21 days treatment. **G** Photos of tumors after 21 days treatment. **H** H&E-stained in tumor regions of different treatment groups by IHC and immunofluorescence analysis of the expression of VEGF, Ki67 and HIF-1α; scale bar = 200 µm

To determine the radiosensitization effect of CeO_2_–MnO_2_ in vivo, HeLa tumor-bearing mice were divided into four groups randomly: Saline, X-ray, CeO_2_–MnO_2_, and CeO_2_–MnO_2_ + X-ray. A schematic illustration of all animal experiments is given in Fig. 5A. Due to the tumor microenvironment, CeO_2_–MnO_2_ decomposes to generate Mn^2+^ with T1 imaging function. Based on the above properties, we investigated the potential of CeO_2_–MnO_2_ in MRI, which can be used to assess in situ drug accumulation in tumor regions. As seen in Fig. 5B, C the T1-weighted signal intensity of tumor sites in mice was significantly enhanced at 2 h after injection, and the signal was strongest at 4 h. This indicates that CeO_2_–MnO_2_ can rapidly penetrate into the tumor and decompose in response to the tumor microenvironment, while the accumulation of CeO_2_–MnO_2_ was highest at 4 h. And with the metabolism of CeO_2_–MnO_2_, the T1 signal gradually diminished. Consequently, these results indicate that CeO_2_–MnO_2_ accumulates rapidly at tumor locations and becomes Mn^2+^, which can be used as a T1 contrast agent to guide tumor treatment in vivo, while this rapid metabolism also enhances biosafety. To investigate the synergistic effect between X-rays and drugs, we constructed a HeLa cell nude mouse subcutaneous tumor model. Tail vein injection of CeO_2_–MnO_2_ and X-rays synergistically kill tumor. During the treatment period, the length and width of the tumor area were measured every 2 days to calculate the tumor volume, and the weight was measured. At the end of 21 days of treatment, the CeO_2_–MnO_2_ combination radiotherapy group had a better treatment effect compared to the other groups. According to Fig. 5D, the body weight of all experimental groups did not fluctuate much during 21 days, which proved that there was no significant toxicity in CeO_2_–MnO_2_ group. Meanwhile, according to Fig. 5E, under the treatment of the CeO_2_–MnO_2_ co-X-ray, the tumor volume was the smallest after 21 days. As shown in Fig. 5F, G, the tumor mass and representative tumor photos of mice clearly showed that the anti-tumor efficiency of CeO_2_–MnO_2_ combined with X-ray treatment was superior to other treatment groups. In addition, H&E staining of tumor tissue sections showed that CeO_2_–MnO_2_ combined with radiotherapy effectively promoted apoptosis in cancer cells (Fig. 5H). To further evaluate the inhibitory effect of treatment on cancer cell proliferation and angiogenesis, immunofluorescence (IF) staining is performed using Ki67 and VEGF antibodies. Representative Ki67 and VEGF in each group are shown in Fig. 5H. At the same time, since CeO_2_–MnO_2_ catalyzes the production of O_2_ by H_2_O_2_, thereby improving tumor hypoxia, the enhanced synergistic therapeutic effect of X-ray and CeO_2_–MnO_2_ in overcoming tumor hypoxia is demonstrated by the expression of HIF-1α (Fig. 5H).

### Biosafety of CeO_2_–MnO_2_ in vivo

**Fig. 6:**
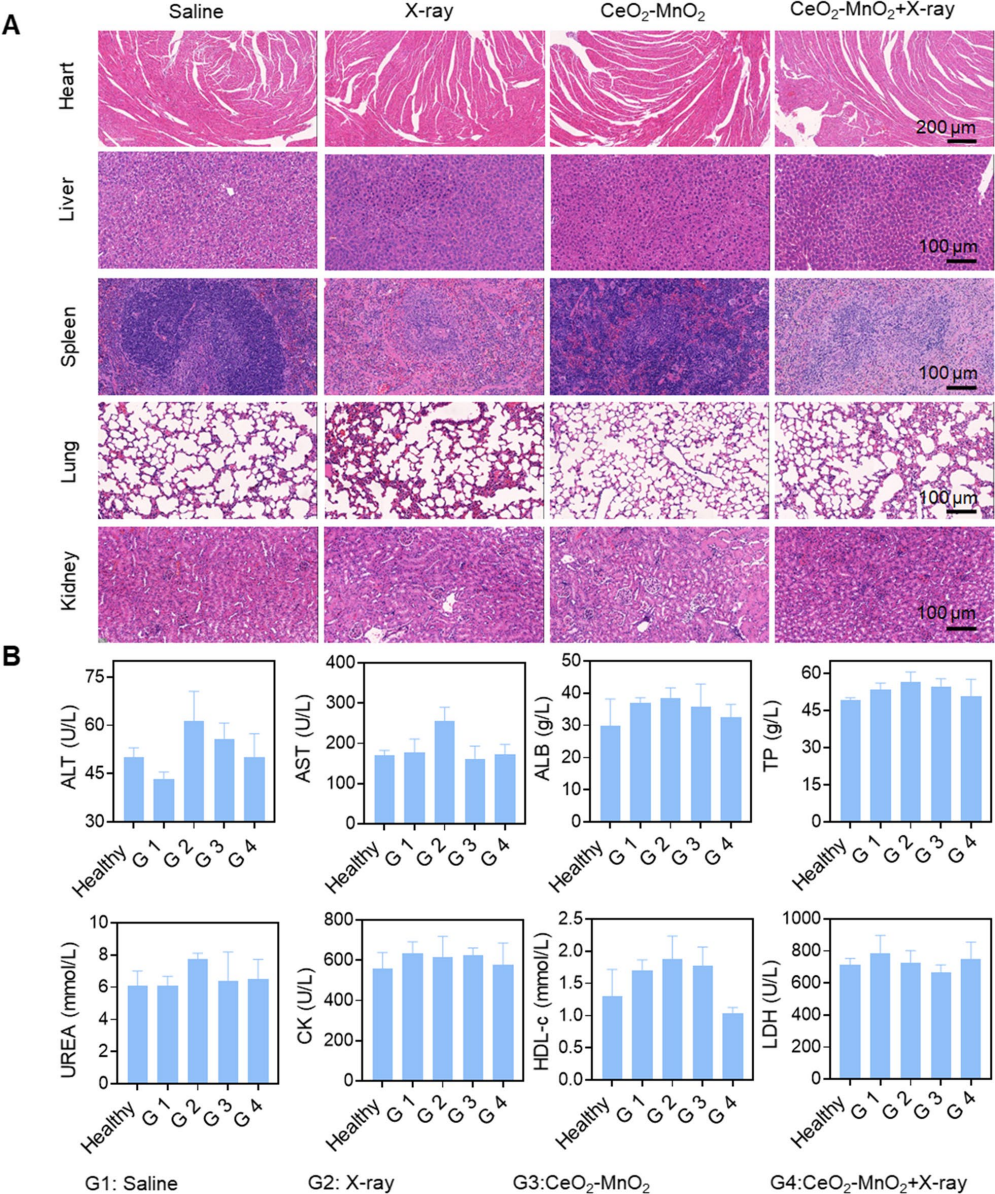
In vivo biosafety of CeO_2_–MnO_2_ nanoparticles. **A** H&E staining of main organs under different treatments after 21 days. **B** Hematological analysis of mice with different treatments for 21 days. G1: saline; G2:X-ray; G3: CeO_2_–MnO_2_; G4: CeO_2_–MnO_2_ + X-ray

We systematically evaluated the potential toxicity of CeO_2_–MnO_2_ in synergistic treatment groups with X-ray, and the microscopic images of the tissues by H&E staining showed that CeO_2_–MnO_2_ combined with X-ray had no significant toxicity to the major organs of mice (Fig. 6A). Blood was also collected to determine biochemical indexes such as ALT, AST, ALB, TP and UREA to evaluate liver, kidney and heart functions. The high safety and low toxicity of CeO_2_–MnO_2_ as a radiosensitizer in cancer treatment was confirmed compared to the healthy group (Fig. 6B). The low toxicity of the nanomedicine in vivo was confirmed, suggesting further biomedical applications of the formulation.

## Conclusion

In conclusion, we synthesized CeO_2_–MnO_2_ nanoparticles and characterized their structures using a series of characterization tools. In addition, CeO_2_–MnO_2_ nanoparticles have anti-tumor properties and can respond to GSH and H_2_O_2_, generating large amounts of ROS and oxygen, enhancing the radiotherapy efficacy and improving the cancer microenvironment. They also have MRI functionality to pinpoint the tumor lesion at the tumor site and improve the anti-tumor effect. These properties enable CeO_2_–MnO_2_ nanoparticles to have significant anti-tumor properties in vivo and in vitro. In summary, we present a radiosensitizer that enhances the radiotherapy efficacy while ensuring low toxicity to normal sites, which will greatly help promote efficient and low toxicity radiotherapy.

## Experimental section

### Materials and methods

Cerous nitrate hexahydrate [Ce(NO_3_)_3_∙6H_2_O], Potassium permanganate (KMnO_4_),Sodium hydroxide (NaOH), Thiazolyl blue tetrazolium bromide (MTT), Propidium iodide (PI) were obtained from Sigma-Aldrich. Dulbecco’s modified Eagle’s medium (DMEM) and fetal bovine serum (FBS) were purchased from Gibco Thermofisher Scientific Inc. Hydrogen peroxide test kits was obtained from Beyotim. Annexin V-FITC/PI Apoptosis Kit was purchased from Dojindo Chemical Technology Co., Ltd (China). 30% H_2_O_2_ solutions was purchased from Guangzhou Chemical Reagent Factory (China).All animal experiments were conducted under the approval of the Animal Experimentation Ethics Committee of JinanUniversity.

### The synthesis of CeO_2_

Ce (NO_3_)_3_∙6H_2_O (1.736 g) was dissolved in 10 mL ultrapure water, sodium hydroxide 19.2 g was dissolved in 70 mL of ultrapure water, and the two solutions were mixed and stirred at room temperature for 30 min. The mixture was heated to 100 °C and refluxed for 24 h. The reaction product was centrifuged at 8000 rpm for 10 min and washed three times with ultrapure water. The precipitate was dried overnight in an oven at 60 ℃. 20 mg of the dried product was dissolved in 10 mL of ultrapure water, sonicated for 2 h until completely dissolved, transferred to a Teflon bottle and calcined in an autoclave at 160 ℃ for 12 h. The product then was extracted from the reaction at 8000 rpm. After the reaction, the product was dried at 60 ℃, and the powder was obtained as CeO_2_.

### The synthesis of MnO_2_

Add 20 mL of 0.1 M MnCl_2_ solution to 1 M NaOH to adjust pH to 10, stir vigorously at room temperature for 2 h, and dialyze the solution for 24 h to obtain MnO_2_ solution. Stir for 2 h. Dialyze the solution for 24 h to obtain MnO_2_ solution.

### The synthesis of CeO_2_–MnO_2_

1.736 g of cerium nitrate hexahydrate was dissolved in 10 mL of ultrapure water and 19.2 g of sodium hydroxide was dissolved in 70 mL of ultrapure water. The two solutions were mixed at room temperature and stirred for 30 min, then the mixture was heated to 100 ℃ and refluxed for 24 h. The product was centrifuged at 8000 rpm for 10 min and washed three times with ultrapure water. The precipitate was dried overnight in an oven at 60 ℃. Add 80 mg of the product to 35 mL of KMnO_4_ solution at a concentration of 0.01 M. Transfer the solution to a 50 mL PTFE vial and calcine in an autoclave at 140 ℃ for 12 h. Centrifuge at 8000 rpm for 10 min and wash three times to give the final product as CeO_2_–MnO_2_.

### CeO_2_–MnO_2_ catalyzes the production of O_2_ from H_2_O_2_ in vitro

After mixing 400 µM, 200 µM and 100 µM H_2_O_2_ with aqueous CeO_2_–MnO_2_ solution thoroughly at room temperature, the concentration of oxygen was measured using a dissolved oxygen meter and the values were recorded for 15 min.

### Detection of the rate of H_2_O_2_ consumption by CeO_2_–MnO_2_ in vitro

The rate of hydrogen peroxide scavenging by CeO_2_, MnO_2_ and CeO_2_–MnO_2_ nanoparticles at 100 µg/mL was detected using the hydrogen peroxide kit, while the rate of hydrogen peroxide scavenging by CeO_2_–MnO_2_ nanoparticles at 10, 20, 40 and 80 µg/mL was detected.

### GSH response of CeO_2_–MnO_2_

GSH of 8 mM, 4 mM, 2 mM, 1 mM and 0.5 mM were applied with CeO_2_–MnO_2_ for 5 min at room temperature, and then the color change of the solution was recorded and the UV–Vis absorption spectrum of the solution was detected.

### Determination of cell viability

The cells involved included human cervical cancer cells, HeLa cells, and human normal hepatocytes cells, MIHA cells. HeLa cells and MIHA cells at the logarithmic growth stage were inoculated in 96-well plates at 3 × 10^4^ cells/mL, 100 µL/well, and incubated with different concentrations of CeO_2_, MnO_2_ and CeO_2_–MnO_2_ for 8 h after 24 h. After irradiation, the cells were incubated in the incubator for 48 h. The cell survival rate was determined by MTT assay.

### ROS level detection

HeLa cells at logarithmic growth stage were inoculated in 96-well plates at a density of 3 × 10^5^ cells/mL, and incubated with the same concentration of CeO_2_, MnO_2_ and CeO_2_–MnO_2_ for 4 h. After incubation, DCFH-DA probe (Ex: 488 nm, Em: 525 nm) and DHE probe (Ex: 300 nm, Em: 610 nm) were added respectively, and incubated at 37 ℃ for half an hour, followed by exposure to 4 Gy and immediate detection of fluorescence intensity values at 5 min intervals using an enzyme marker.

### Cell cycle and apoptosis assays

To demonstrate the apoptosis and cycle ratio of CeO_2_–MnO_2_ in HeLa cells, the assay was analyzed using flow cytometry. HeLa cells at logarithmic growth stage were inoculated in 6 cm dishes at a density of 8 × 10^4^ cells/mL, and incubated with the same concentration of CeO_2_, MnO_2_ and CeO_2_–MnO_2_ for 4 h after 24 h. After exposure to 4 Gy radiation and continued incubation for 48 h, cells were collected and stained with PI for 15 min, filtered, and assayed for cell cycle. Similarly, logarithmic growth phase HeLa cells were inoculated in 6-well plates at a density of 1 × 10^5^ cells/mL overnight, and after the cells were plastered, the same concentrations of CeO_2_, MnO_2_ and CeO_2_–MnO_2_ were added and incubated for 6 h. The cells were exposed to 4 Gy radiation and continued to be incubated for 48 h. The cells were collected and stained with PI and Annexin V for 15 min to detect the percentage of apoptosis.

### Cellular localization experiments

The lysosomes and nuclei were stained and incubated with the same concentration of coumarin-6-labeled CeO_2_–MnO_2_ for 0 h, 1 h, 2 h, 4 h, 8 h and 12 h. The medium was removed and gently washed several times with PBS, and the fluorescence signal of the intracellular drug was recorded under a fluorescence microscope.

### Cloning experiments

HeLa cells were inoculated in 6-well plates (2000 cells per well) and incubated in a humid CO_2_ incubator for 24 h. After complete cell adhesion, cells treated with 40 μg/mL of CeO_2_–MnO_2_ were co-incubated for 6 h and irradiated with X-ray radiation. 7 days later, the post-treated cells were washed with PBS, immobilized with paraformaldehyde, and then stained with 10% crystalline violet. The corresponding digital photographs were recorded and cell survival rates were calculated based on relativity analysis.

### Tumor modeling

Female BALB/c-nude mice were purchased at 4 weeks of age from Beijing Vital River Laboratory Animal Technology Co., Ltd. After the quarantine period, when the mice reached 18–20 g, they were inoculated subcutaneously with 100 μL of HeLa cells at a density of 1 × 10^7^ cells/mL. After the quarantine period, when the mice reached 18–20 g, 100 μL of HeLa cells at a density of 1 × 10^7^ cells/mL were inoculated subcutaneously, and when the tumor volume grew to 120–150 mm^3^, the mice were randomly grouped to start the next step of the experiment.

### Study on the antitumor activity in vivo

4 groups were randomly grouped, with 4 mice in each group (1) Blank control group: 100 µL of saline in the tail vein (2) X-ray group: 100 μL of saline in the tail vein (3) CeO_2_–MnO_2_ group: 2 mg/kg (4) CeO_2_–MnO_2_ + X-ray group: 2 mg/kg after tail vein dosing. The mice were irradiated with 4 Gy, and the total radiation dose was 40 Gy. The tumor volume was calculated by measuring the length and width of the tumor every 2 days, and the weight of the mice was recorded. 21 days later, the mice were subjected to blood sampling from the orbital plexus, and the tumor body and major organs were removed.

### In vivo MR imaging

Homozygous BALB/c nude mice were injected with 10 mg/kg of CeO_2_–MnO_2_ solution in the tail and MR imaging was performed using MR imaging system.

### Statistics analysis

Data are expressed as mean ± standard deviation. Calculation and analysis of all experimental results using GraphPad Prism 8.0. A two-tailed Student's t-test was applied to determine the statistical significance of the differences between the two groups, and variances between multiple groups were tested using the ANOVA. The difference from *P* < *0.05* (*) or *P* < *0.01* (**) is considered statistically significant.

## Supplementary Information

**Additional file 1: Figure S1**. The average size of CeO_2_, MnO_2_ and CeO_2_–MnO_2_. **Figure S2**. Rates of hydrogen peroxide scavenging by CeO_2_, MnO_2_ and CeO_2_–MnO_2_ under 100 µg/mL. **Figure S3**. •O_2_^-^ level of CeO_2_, MnO_2_ and CeO_2_–MnO_2_ under different X-rays (4 Gy). **Figure S4**. ROS level of CeO_2_, MnO_2_ and CeO_2_–MnO_2_ under different X-rays (4 Gy). **Figure S5 **. Isobologram analysis of the synergistic antiproliferative effect of the combined application of X-ray and CeO_2_–MnO_2_ on HeLa cells.

## Data Availability

Additional file is available online.
