# Supplementary material for: Alleviating the hypoxic tumor microenvironment with MnO2-coated CeO2 nanoplatform for magnetic resonance imaging guided radiotherapy
Source: J Nanobiotechnology. 2023 Mar 15;21:90. doi: 10.1186/s12951-023-01850-1 (PMC10018832; doi:10.1186/s12951-023-01850-1)
Supplement: Supplementary file 1 — Additional file 1: Figure S1. The average size of CeO2, MnO2 and CeO2–MnO2. Figure S2. Rates of hydrogen peroxide scavenging by CeO2, MnO2 and CeO2–MnO2 under 100 µg/mL. Figure S3. •O2- level of CeO2, MnO2 and CeO2–MnO2 under different X-rays (4 Gy). Figure S4. ROS level of CeO2, MnO2 and CeO2–MnO2 under different X-rays (4 Gy). Figure S5 . Isobologram analysis of the synergistic antiproliferative effect of the combined application of X-ray and CeO2–MnO2 on HeLa cells. [file 12951_2023_1850_MOESM1_ESM.docx]

Additional file

for

Alleviating the hypoxic tumor microenvironment with MnO_2_-Coated CeO_2_ Nanoplatform for magnetic resonance imaging guided radiotherapy

Fen Pi^a#^, Xuanru Deng^a#^, Qian Xue^a^, Lan Zheng^a^, Hongxing Liu^a,b^*, Fang Yang^a^*, Tianfeng Chen^a^

**Figure S1** The average size of CeO_2_, MnO_2_ and CeO_2_-MnO_2_

**Figure S2** Rates of hydrogen peroxide scavenging by CeO2, MnO2 and CeO_2_-MnO_2_ under 100 µg/mL.

**Figure S3** •O_2_^-^ level of CeO_2_, MnO_2_ and CeO_2_-MnO_2_ under different X-rays (4 Gy)

**Figure S4** ROS level of CeO_2_, MnO_2_ and CeO_2_-MnO_2_ under different X-rays (4 Gy).


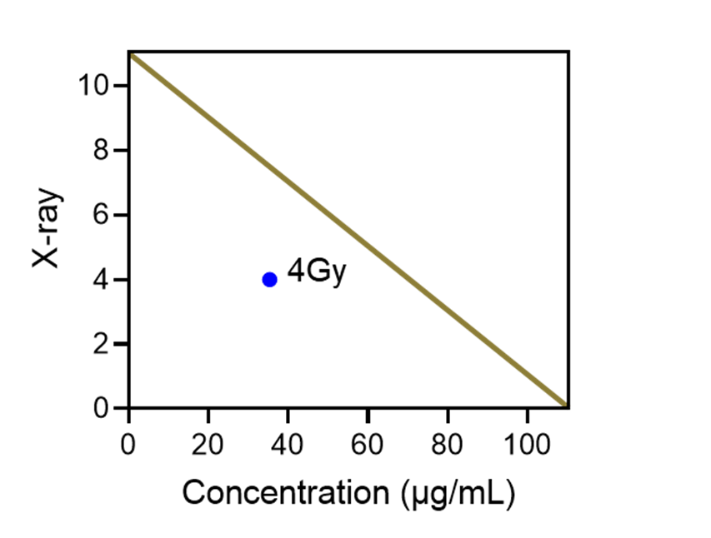


**Figure S5** Isobologram analysis of the synergistic antiproliferative effect of the combined application of X-ray and CeO_2_-MnO_2_ on HeLa cells.
